# Supplementary material for: Evolution in the Debian GNU/Linux software network: analogies and differences with gene regulatory networks
Source: J R Soc Interface. 2020 Feb 12;17(163):20190845. doi: 10.1098/rsif.2019.0845 (PMC7061711; doi:10.1098/rsif.2019.0845)
Supplement: Supplementary Information [file rsif20190845supp1.pdf]

Supplementary Information for “Evolution in the Debian GNU/Linux software network: analogies and differences with genetic regulatory networks”

Pablo Villegas[1,2], **Miguel A. Muñoz**[1], **and Juan A. Bonachela**[3,4,*]

[1]Departamento de Electromagnetismo y Física de la Materia e Instituto Carlos I de Física Teórica y Computacional. Universidad de Granada, E-18071 Granada, Spain
[2]Istituto dei Sistemi Complessi, CNR, via dei Taurini 19, 00185 Rome, Italy
[3]Marine Population Modeling Group, Department of Mathematics and Statistics, University of Strathclyde, Glasgow, G1 1XH, Scotland, UK
[4]Department of Ecology, Evolution, and Natural Resources, Rutgers University, New Brunswick, New Jersey, United States of America
[*]To whom correspondence should be addressed: juan.bonachela@rutgers.edu

SI-1: Properties of the Debian networks

The growth of the total number of packages in the Debian/GNU Linux operating system across its first 14 releases is summarized in Figure S1. The number of packages increased exponentially over time showing two different trends, with the 8th release as the transition point.

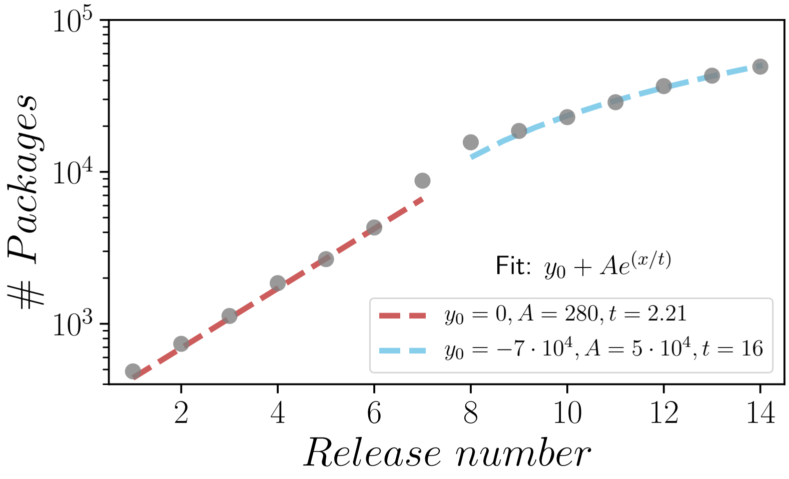

Figure S1. Total number of packages versus release number in Debian GNU/Linux networks. Two different exponential growths can be appreciated: One before release number 8 (red dashed line), and another one thereon (blue dashed line).

SI-2: Probability distributions of Debian networks

We also monitored the out-degree distribution by measuring the cumulative probability distribution for a package to be a requirement for k_{out} packages for each distribution. As shown in Fig.S2 the cumulative version of the out-degree distribution follows a power law, $P(k_{out}) \sim k_{out}^{-\alpha+1}$ with characteristic exponent α. As can be seen, the power law of the out-degree distribution shows an exponent very close to $\alpha = 2$ for all releases (red points represents the probability distributions and black lines are fits with an exponent is indicated for each case).

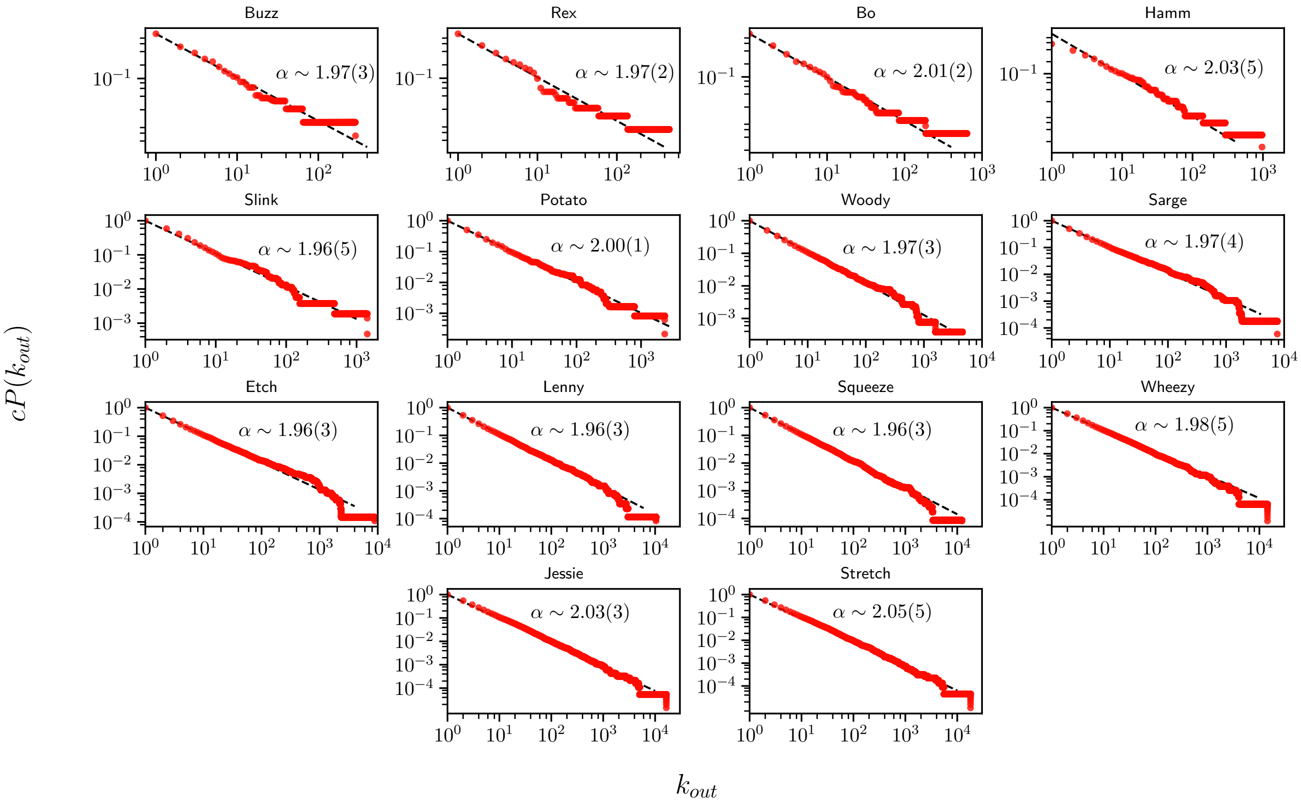

Figure S2. Cumulative degree distribution for outgoing dependencies for all releases (red points) and associated fits (black dashed lines).

On the other hand, the probability for a package to depend on k_{in} packages follows a stretched exponential, $P(k_{in}) \sim \exp(-(\frac{k_{in}}{\tau})^{\beta})$ with characteristic exponent β. The exponent of the stretched exponential for the in-degree distributions decays from $\beta = 2$ (normal distribution) to an approximately-stationary $\beta \approx 0.5$ after the 8th release (blue points).

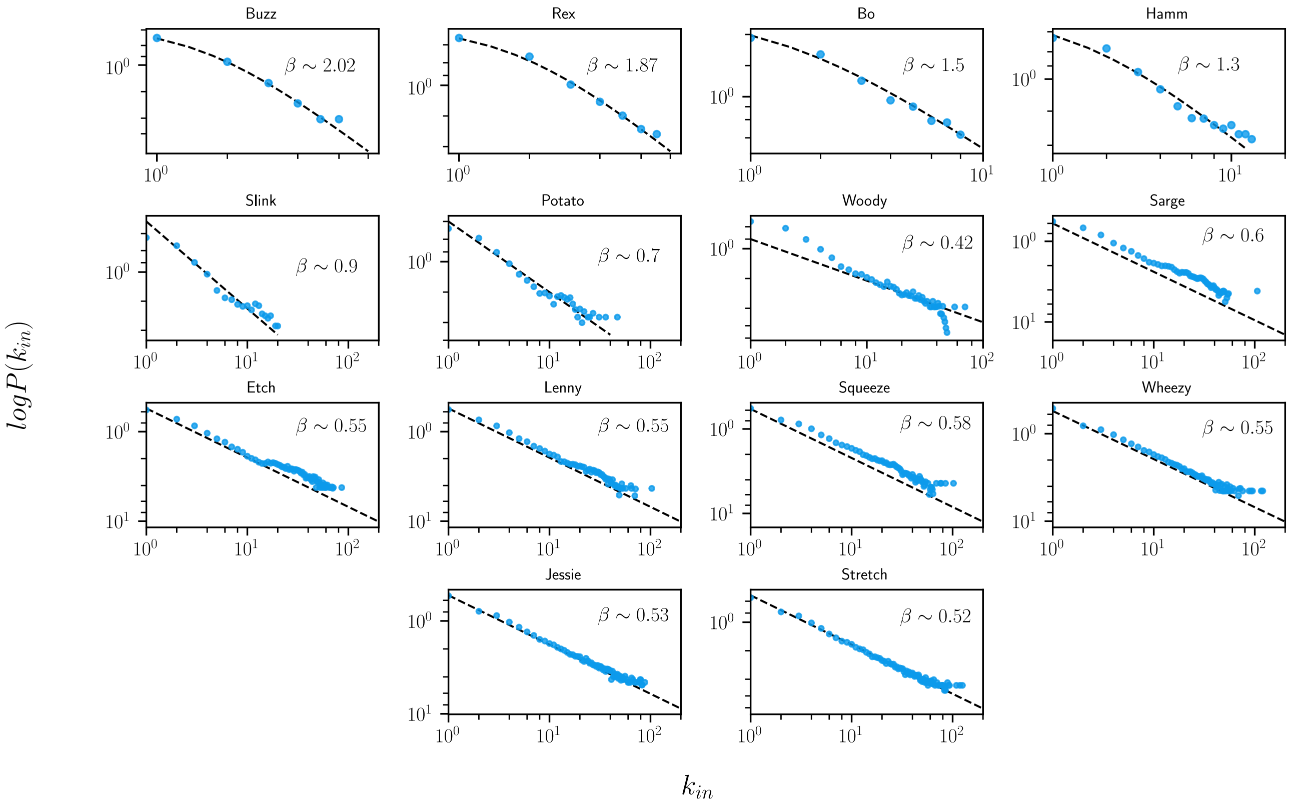

Figure S3. Logarithm of the degree distribution for incoming dependences for all releases (blue points, note the inversion of the vertical axis), showing the change from a half-normal distribution decay ($\beta = 2$) for the first version to a stretched exponential distribution ($\beta = 0.5$) for the last one. Dashed black lines are fits of the form $logP(k_{in}) \sim k^{\beta} + h$.

SI-3: Measures in Debian networks

We also computed the Z-Score with respect to random networks respecting the degree sequence of the network (i.e. the number of in and out neighbors for each package). The Z-score of an observable for each network is defined as the difference between the observable and its mean across randomizations, taking into account its variability (i.e. normalizing by the standard deviation). Mathematically, $z = \frac{x-\mu}{\sigma}$. Figure S4 shows the Z-Score values for the modularity index (Z-Score Q) and the average path length (Z-score l_G). Both values are far from $Z \sim 0$, indicating deviations on the order of $(500-800)\sigma$ for the modularity index in the last distributions (i.e. the networks show a modularity index much larger than expected in the random case), and on the order of $(10-30)\sigma$ for the average path length in the last distributions (i.e. the networks show an average path length much shorter than expected in the random case). In addition, Fig. S5 shows the growth of the average path length as a function of the logarithm of the total number of packages. For Debian networks, and as expected for small-world networks, l_G exhibits a growth proportional to the logarithm of the size of the network, i.e. $log(N_{pack})$. As stated in the main text, there exists a shift in behavior –around the 8th distribution– where all the major changes take place.

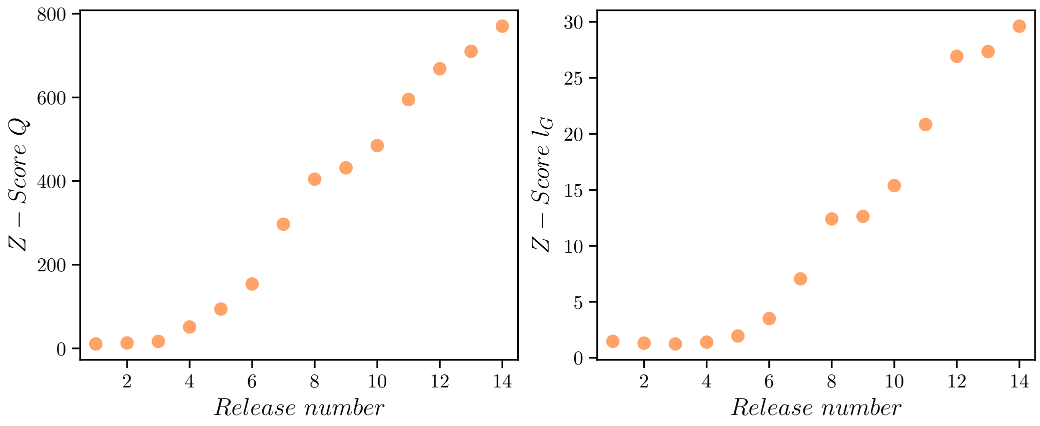

Figure S4. Left: Z-score value for the modularity index versus release number; modularity shows a sustained increase over time with respect to the expected value of the "swapped" network. Right: Z-score value for the average path length versus release number; the average path length remains much lower than expected as the system size increases in relation to the "swapped" networks.

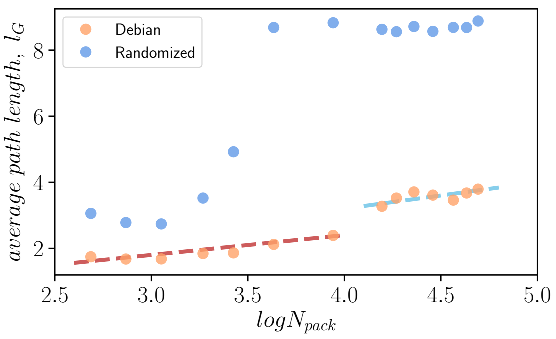

Figure S5. Average path length (l_G) for the original Debian networks (orange points) and the "swapped" networks (blue points) as a function of the log-number of packages. Dashed lines (red and blue) stand for different growths proportional to $log(N_{pack})$, before and after the 8th distribution.

SI-4: Probability distributions of gene regulatory networks

We also measured the cumulative probability distribution for regulated genes in diverse gene regulatory networks (GRNs) for which data were publicly available (see main text). As shown in Fig.S6, the cumulative version of the out-degree distribution follows a power law $P(k_{out}) \sim k_{out}^{-\alpha+1}$ with characteristic exponent α, at least for a few decades. The associated exponents are in the range $\alpha \in (1.5-2)$.

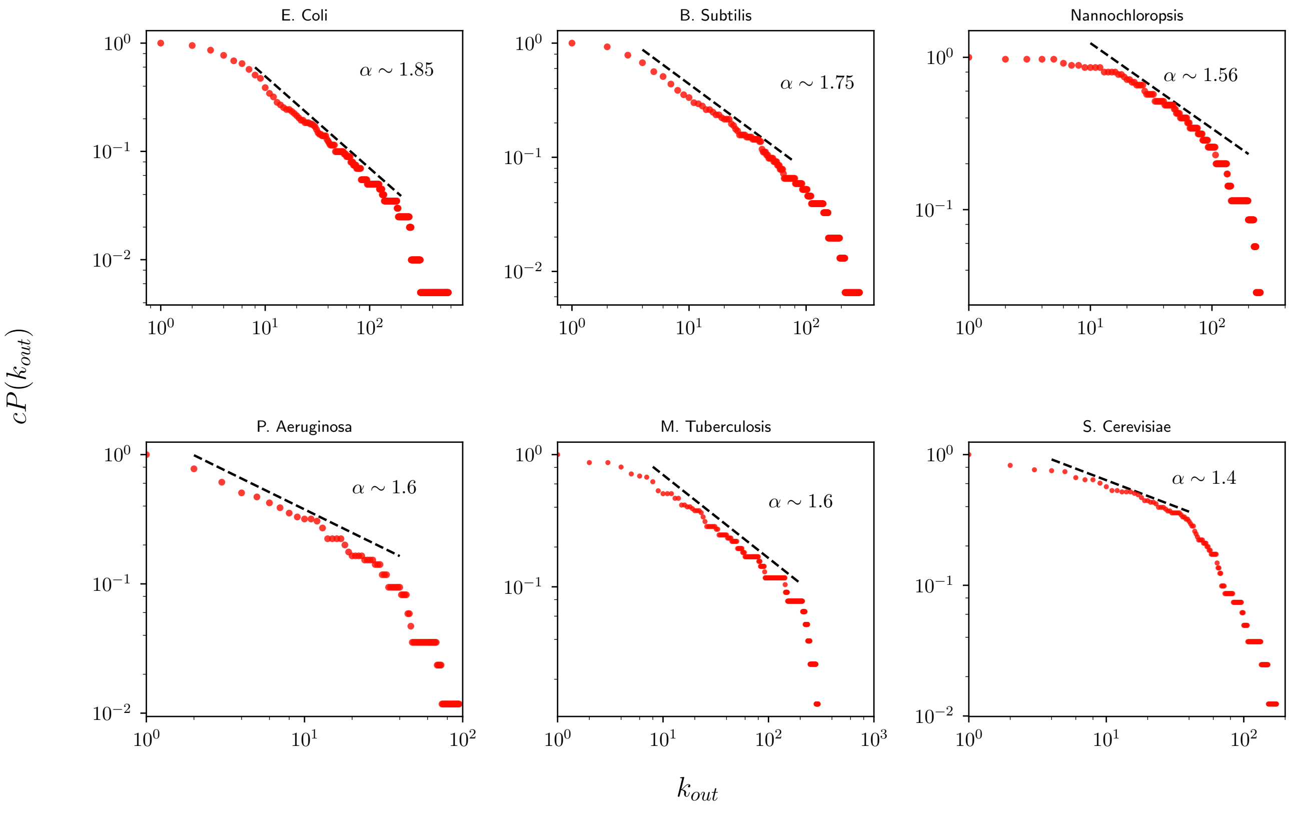

Figure S6. Cumulative degree distribution for regulated (i.e. outgoing) genes for different GRNs (red points), with guides to the eye for the power-law fit (black dashed lines).

On the other hand, the probability for a gene to be regulated by k_{in} genes follows a stretched exponential, $P(k_{in}) \sim \exp(-(\frac{k_{in}}{\tau})^{\beta})$ with characteristic exponent β (see Fig. S7). The exponent of the stretched exponential follows $\beta = 1$ (i.e. exponential distribution) except for $E.Coli$, for which $\beta \approx 0.67$.

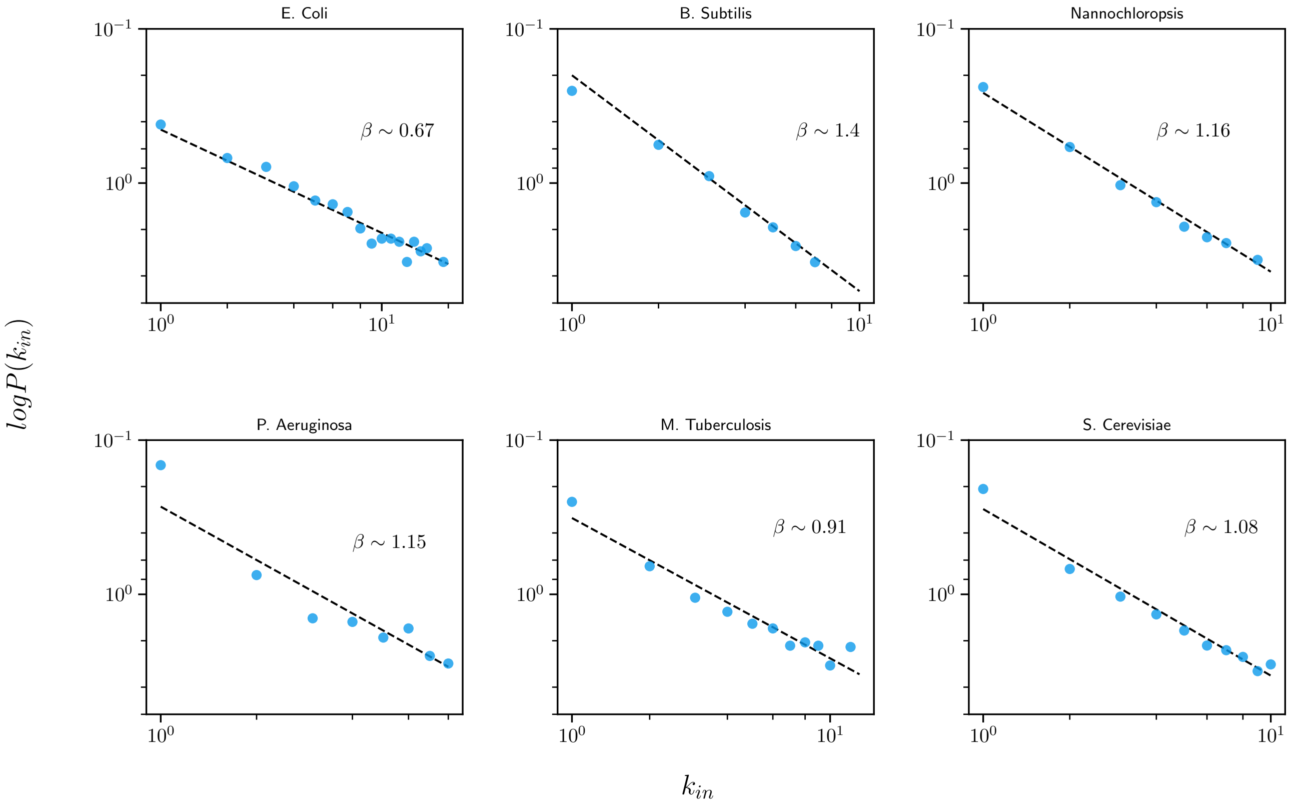

Figure S7. Cumulative degree distribution for regulatory (i.e. incoming) genes in different gene regulatory networks (blue points, note the inverted vertical axis), with guides to the eye for the exponential fit (black dashed lines).

SI-5: Further comparison between GRNs and Debian networks

Gene knock-out experiments silencing individual genes allow for a measure of the "vulnerability" (see main text) of GRNs by monitoring the cascade of metabolic changes that are affected by such a network-state change. As shown in Figure S8, the distribution of the size of such cascade follows a power-law distribution with an exponent $\tau \sim 3/2$, similar to that measured for the Debian networks. See main text for further details.

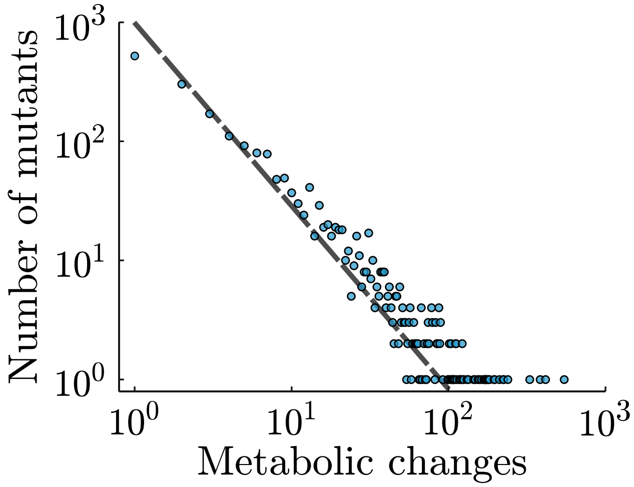

Figure S8. Distribution of metabolic changes in gene knockout mutants for E.Coli (blue points). Adapted from Fuhrer et al[1]. The dashed line is a power law with exponent $\tau = \frac{3}{2}$, as guide to the eye.

References

1. Fuhrer, T., Zampieri, M., Sévin, D. C., Sauer, U. & Zamboni, N. Genomewide landscape of gene–metabolome associations in escherichia coli. *Mol. Sys. Biol.* **13**, 907 (2017).
